# The Biological Clock is Regulated by Adrenergic Signaling in Brown Fat but is Dispensable for Cold-Induced Thermogenesis

**DOI:** 10.1371/journal.pone.0070109

**Published:** 2013-08-26

**Authors:** Siming Li, Qi Yu, Guo-Xiao Wang, Jiandie D. Lin

**Affiliations:** Life Sciences Institute and Department of Cell and Developmental Biology, University of Michigan, Ann Arbor, Michigan, United States of America; Pennsylvania State University, United States of America

## Abstract

The biological clock plays an important role in integrating nutrient and energy metabolism with other cellular processes. Previous studies have demonstrated that core clock genes are rhythmically expressed in peripheral tissues, including the liver, skeletal muscle, pancreatic islets, and white and brown adipose tissues. These peripheral clocks are entrained by physiological cues, thereby aligning the circadian pacemaker to tissue functions. The mechanisms that regulate brown adipose tissue clock in response to physiological signals remain poorly understood. Here we found that the expression of core clock genes is highly responsive to cold exposure in brown fat, but not in white fat. This cold-inducible regulation of the clock network is mediated by adrenergic receptor activation and the transcriptional coactivator PGC-1α. Brown adipocytes in mice lacking a functional clock contain large lipid droplets accompanied by dysregulation of genes involved in lipid metabolism and adaptive thermogenesis. Paradoxically, the “clockless” mice were competent in maintaining core body temperature during cold exposure. These studies elucidated the presence of adrenergic receptor/clock crosstalk that appears to be required for normal thermogenic gene expression in brown fat.

## Introduction

Obesity and its associated metabolic disorders have become a global epidemic that elevates the risk for type 2 diabetes, cardiovascular disease, and non-alcoholic fatty liver disease. Obesity results from a chronic excess of energy intake over energy expenditure. In rodents, brown adipose tissue (BAT) plays an important role in adaptive thermogenesis that maintains core body temperature in cold environment and contributes to whole body energy homeostasis [1], [2]. This unique function of BAT is attributed to its high oxidative capacity and the expression of uncoupling protein 1 (UCP1), which dissipates mitochondrial proton gradient through heat production. Recent studies have demonstrated that functional brown fat is present in a subset of adult humans and can be activated by cold exposure and adrenergic agonists [3], [4], [5], [6]. Unlike BAT, white adipose tissue (WAT) has relatively low mitochondrial content and serves as a major reservoir for fuel storage. Remarkably, clusters of UCP1-positive brown adipocyte-like cells emerge in white fat depots in response to chronic cold exposure or adrenergic stimulation [7], [8], [9]. These “beige” adipocytes originate from progenitor pools that are developmentally distinct from brown adipocytes in BAT [10], [11]. Several transcriptional factors and cofactors have been identified to regulate different aspects of brown fat development, including *Prdm16, C/EBPβ, Foxc2, Twist1, PGC-1α* and *PGC-1β* [10], [12], [13], [14], [15], [16], [17], [18].

In mammals, the circadian clock is responsible for synchronizing physiology and behaviors with the environment [19], [20], [21]. In fact, core body temperature fluctuates according to light/dark cycles that may impinge on the rhythmicity of the clock itself. Indeed, recent work has demonstrated that daily temperature fluctuations as small as 1.5°C can maintain rhythmic gene expression in several mammalian cell types and mouse tissues [22], [23], [24], [25]. The synchronization of circadian timing and biological processes is largely mediated through the molecular clock, which consists of transcriptional activators and repressors assembled into negative feedback loops. Brain and muscle Arnt-like 1 (*Bmal1*) forms heterodimers with *Clock* or Neuronal PAS domain-containing protein 2 (*Npas2*) and drives the expression of transcriptional repressors, including Cryptochrome (*Cry*), Period (*Per*) and *Rev-erbα* [26], [27], [28]. The function of *Bmal1* is regulated at the transcriptional level by the *ROR/PGC-1α* activator complex and also at the posttranslational level by modifications, such as acetylation [29], [30], [31]. Recent studies have demonstrated that the components of the clock oscillator are expressed in diverse peripheral tissues, including the liver, skeletal muscle, pancreatic islets, white and brown adipose tissues [32], [33], [34], [35], [36], [37], [38]. Further, the phase of liver clock is particularly sensitive to meal timing [39]. While robust rhythms of clock gene expression have been observed in brown and white fats, the nature of physiological signals that impinge on adipose tissue clocks remain largely undefined.

In this report, we demonstrated that the expression of core clock genes is highly induced by cold exposure in brown fat, but not in white fat, a process mediated by the activation of adrenergic receptor signaling and the transcriptional coactivator *PGC-1α*. Mice lacking a functional clock displayed abnormal brown fat development and altered expression of genes involved in lipid metabolism and adaptive thermogenesis. Paradoxically, the “clockless” mice were competent in maintaining their core body temperature during cold exposure, likely due to a compensatory increase in UCP1 expression and activation of muscle thermogenesis through upregulation of Sarcolipin. These studies illustrate the presence of sympathetic nervous system/clock crosstalk in brown fat.

## Results

### Regulation of brown fat clock by adrenergic signaling

**Figure 1:**
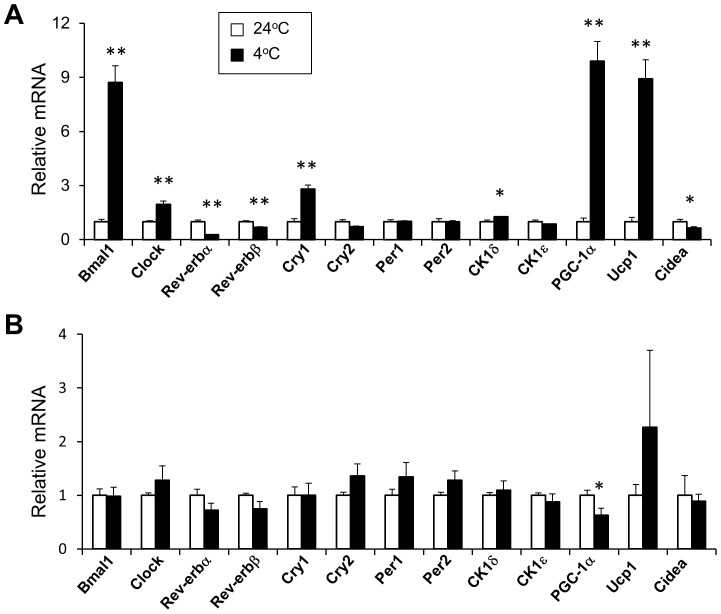
Regulation of clock gene expression in adipose tissues by cold exposure. Mice were maintained at ambient temperature (24°C, open, n = 5) or exposed to cold (4°C, filled, n = 4) for 5 hours. qPCR analyses of gene expression in BAT (A) and WAT (B). Data represent mean ± SEM, 24°C vs. 4°C, *p<0.05. **p<0.01.

The biological clock plays an integral role in temporal regulation of energy metabolism and other cellular processes. Previous studies have demonstrated that clock genes are rhythmically expressed in white and brown adipose tissues [32], [36], [37]. However, whether the adipose tissue clocks respond to adrenergic signals and participate in adaptive thermogenesis has not been explored. To address this, we measured clock gene expression in mouse adipose tissues following acute cold exposure. As expected, the expression of *PGC-1α* and *Ucp1* in brown fat was markedly increased in response to cold exposure (Fig. 1A). While mRNA levels of *Bmal1, Clock, Cry1* and casein kinase 1δ (*CK1δ*) were significantly increased, the expression of *Rev-erbα* and *Rev-erbβ*, nuclear hormone receptors that repress the transcription of target genes, including *Bmal1*, was decreased by cold stress. The expression of other clock genes, such as *Cry2*, *Per1*, *Per2*, and *CK1ε*, was unaltered by cold exposure (Fig. 1A). In contrast, clock gene expression in white fat remained similar before and after cold exposure (Fig. 1B), indicating that the regulation of clock genes by environmental temperature is a unique property of brown adipocytes.

**Figure 2:**
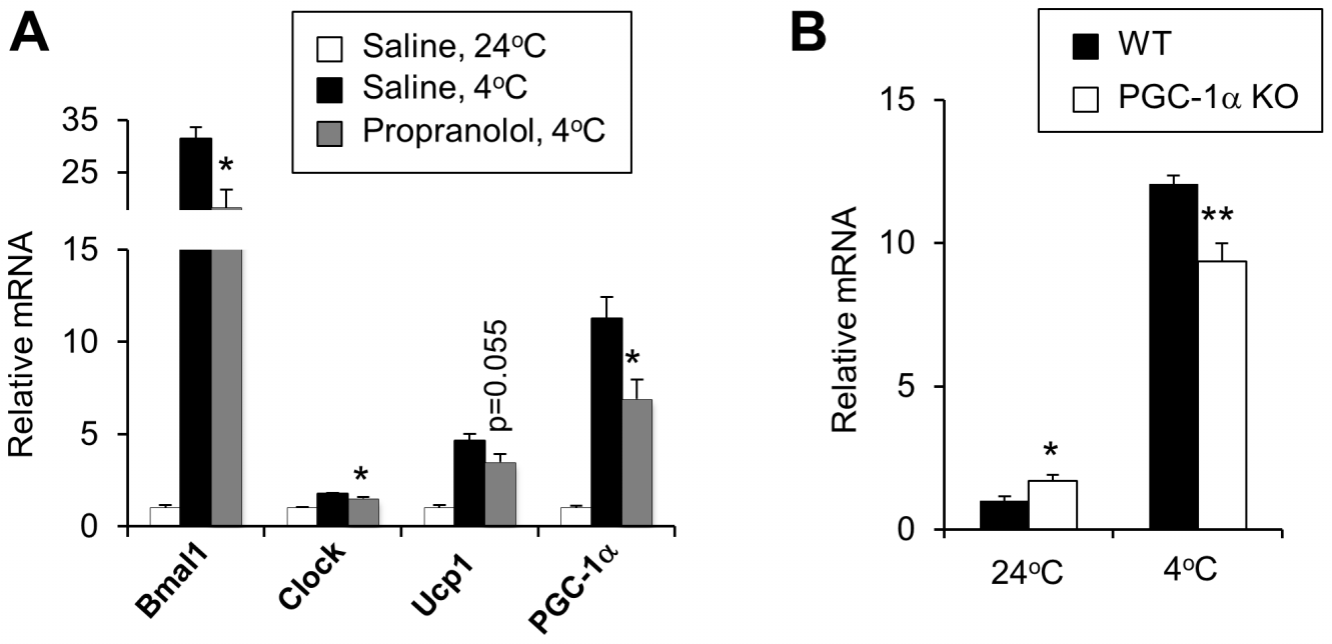
Requirements of β-adrenergic signaling and *PGC-1α* in *Bmal1* expression. A. qPCR analyses of BAT gene expression in saline-injected mice housed at room temperature (24°C, n = 4) and mice treated with saline (filled, n = 4) or propranolol (grey, n = 4) after 3 hrs of cold exposure. Data represent mean ± SEM, saline vs. propranolol at 4°C, *p<0.05. B. *Bmal1* mRNA expression in WT (filled) and *PGC-1α* null (open) mouse brown fat. Mice were housed at ambient temperature (n = 4 per WT or KO group) or subjected to cold exposure for 3.5 hrs (n = 6 per WT or KO group). Data represent mean ± SEM, WT vs. KO, *p<0.05. **p<0.01.

Activation of adrenergic signaling is a major mechanism through which the sympathetic nervous system engages brown fat in response to the cold environments. To determine whether adrenergic signaling mediates the regulation of clock genes in BAT, we treated mice with propranolol, a non-selective β-blocker, and subjected them to cold exposure. Gene expression analyses revealed that cold-inducible expression of *Bmal1, Clock* and *PGC-1α* in BAT were significantly blunted by propranolol treatments (Fig. 2A). The induction of *Ucp1* mRNA was also diminished, although the data only achieved borderline significance. *PGC-1α* is a transcriptional coactivator that regulates mitochondrial oxidative metabolism and has been shown to be required for cold-induced thermogenesis [15], [40]. In hepatocytes, *PGC-1α* also induces *Bmal1* expression through coactivation of the ROR family of orphan nuclear receptors [30]. To determine whether *PGC-1α* mediates the induction of *Bmal1* in brown fat, we examined its expression in wild-type (WT) and *PGC-1α* null mouse brown adipose tissues following cold exposure. Interestingly, baseline *Bmal1* mRNA expression was elevated in *PGC-1α* deficient BAT. However, its induction following cold exposure was diminished when *PGC-1α* is absent (Fig. 2B). These results suggest that β-adrenergic signaling and PGC-1α are required for the induction of *Bmal1* and *Clock* in response to cold exposure.

**Figure 3:**
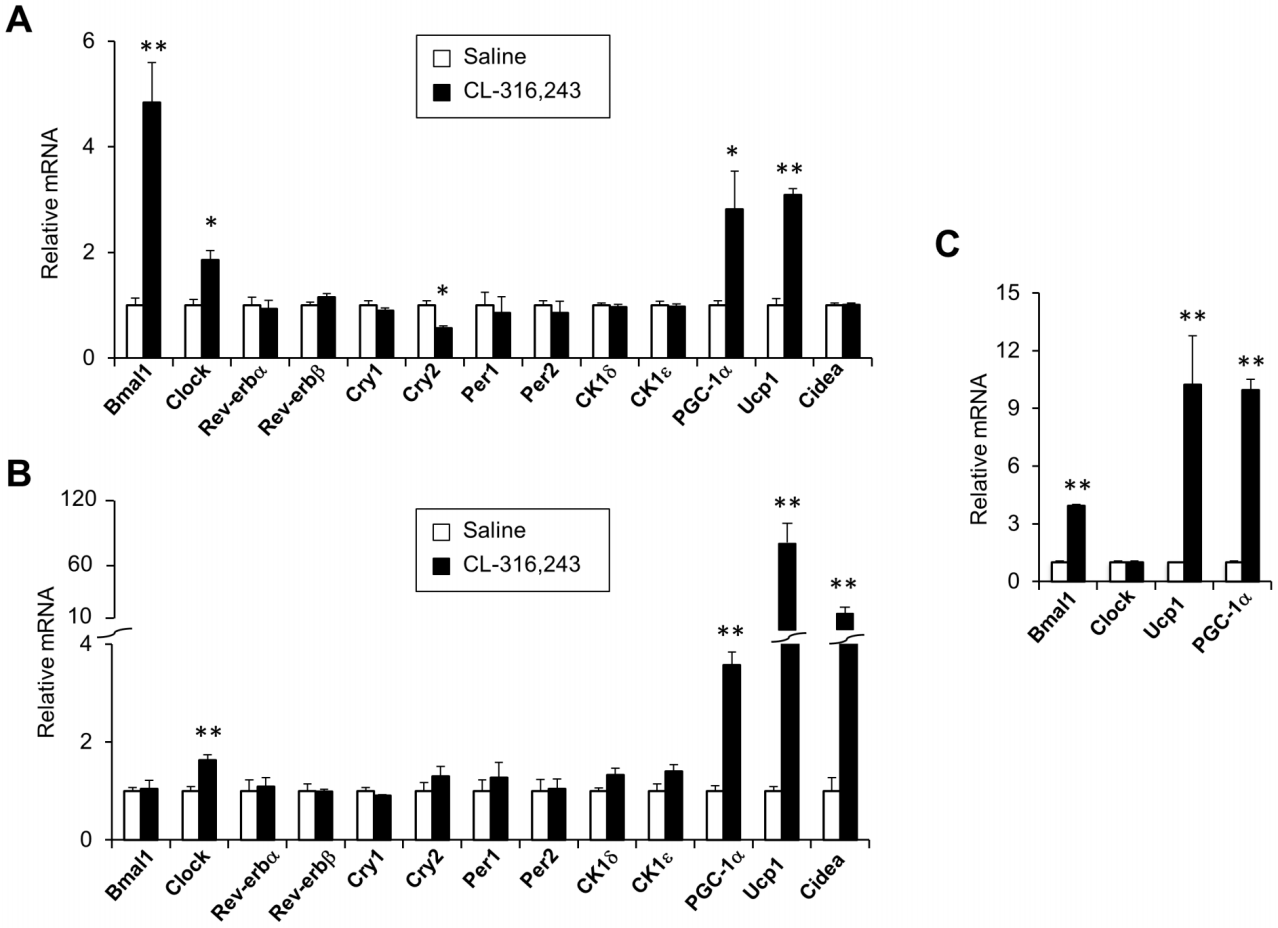
Regulation of clock genes by adrenergic signaling. Three-month old male mice were injected daily with saline (n = 4, open) or CL-316,243 (n = 4, filled) for 7 days. qPCR analyses of gene expression in BAT (A) and WAT (B). Data represent mean ± SEM, saline vs. CL-316,243, *p<0.05, **p<0.01. C. qPCR analysis of gene expression in brown adipocytes 7 days after differentiation. Cells were treated with vehicle (open) or 1 µM norepinephrine (NE, filled) for 5 hrs. Data are collected from three replicates and represent mean ± stdev, vehicle vs. NE, **p<0.01.

To investigate whether activation of adrenergic signaling regulates clock gene expression, we treated mice with CL-316,243, a highly selective β3-adrenergic agonist, by intraperitoneal injection. Treatments of mice with CL-316,243 resulted in elevated expression of *PGC-1α* and *Ucp1* in brown fat. The expression of *Bmal1* and *Clock*, core components of the positive loop of the molecular clock, was also significantly increased by the activation of β3-adrenergic receptor (Fig. 3A). As expected, CL-316,243 treatments induced brown-like adipocytes formation in white adipose tissue, as shown by increased expression of *PGC-1α, Ucp1*, and *Cidea*. However, *Bmal1* gene expression remained unchanged in white adipose tissue following these treatments (Fig. 3B). Interestingly, *Clock* expression is significantly elevated under these conditions. The induction of *Bmal1* expression by adrenergic signaling appeared to be cell-autonomous, as similar observation was obtained in cultured brown adipocytes when treated with norepinephrine (Fig. 3C). We conclude from these studies that clock genes are regulated by the sympathetic nervous system through its stimulation of adrenergic signaling in brown adipocytes.

### A functional clock is required for normal lipid metabolism in brown fat

**Figure 4:**
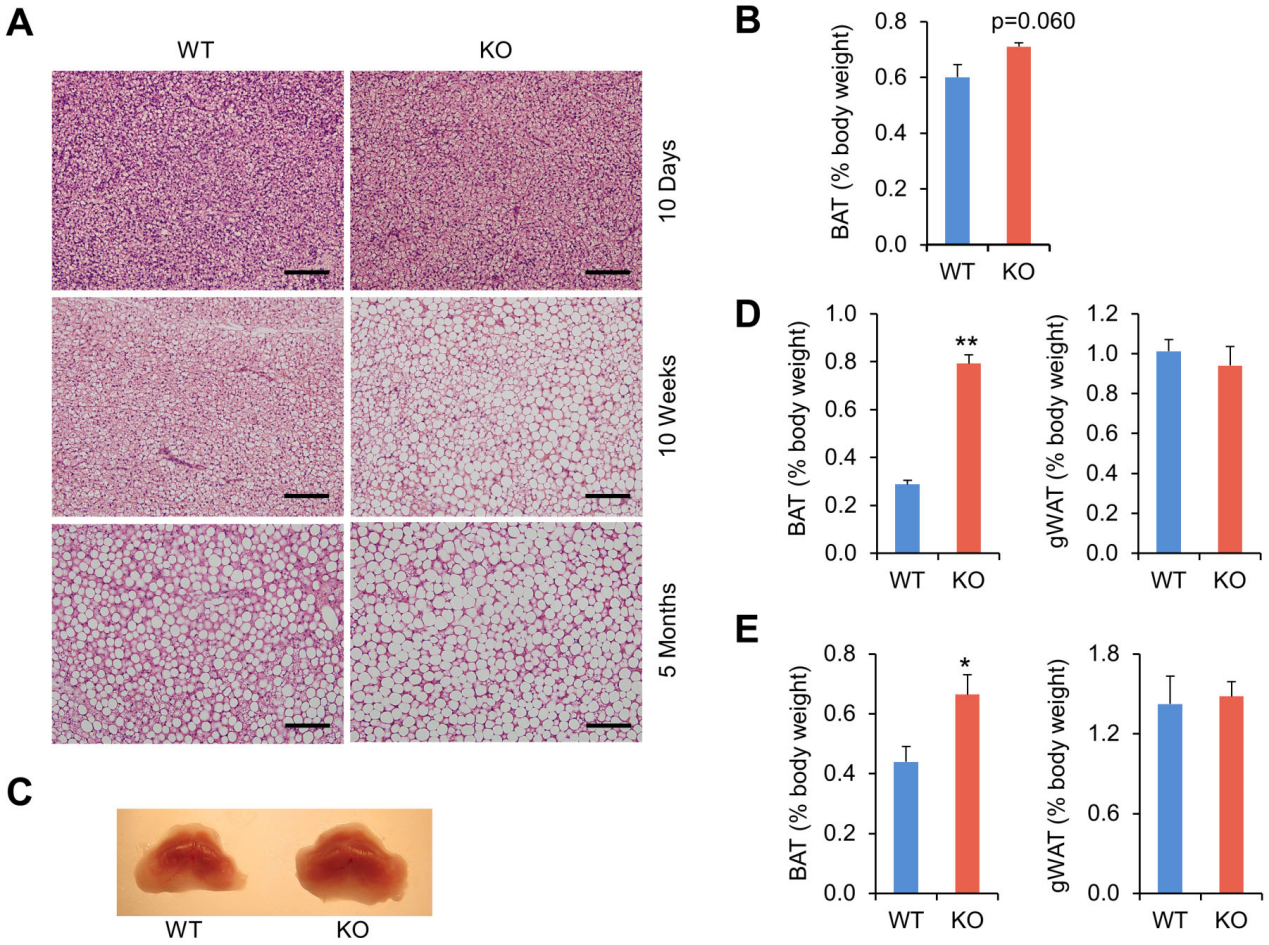
Morphology and histology of brown fats from control and *Bmal1* null mice. A. H&E staining of BAT from WT and *Bmal1* KO mice at 10 days, 10 weeks, or 5 months of age. The bar represents 100 µm. B. BAT/body weight ratio in 10-day old pups. C. Appearance of brown adipose tissues from 10-week old WT and KO mice. D–E. BAT/body weight and gWAT/body weight ratio in 10-week (D) or 5-month (E) old mice. Data represent mean ± SEM, WT (n = 4–6) vs. KO (n = 4–6), *p<0.05, **p<0.01.

Previous studies have shown that mice deficient in *Bmal1* have severely disrupted clock function [41]. To determine the role of clock in brown fat development, we examined brown fat development and function in wild type and *Bmal1* null mice. Brown fat mass in Bmal1 null pups was slightly increased on postnatal day 10. Brown adipocytes appeared largely indistinguishable in control and knockout groups at this stage (Fig. 4A and B). At 10 weeks and 5 months of age, brown fat/body weight ratio was significantly higher in *Bmal1* null mice (Fig. 4C–E). In contrast, gonadal WAT (gWAT)/body weight ratio was similar between control and KO mice. Histological staining indicated that brown adipocytes deficient in *Bmal1* had increased lipid accumulation and larger lipid droplets (Fig. 4A). Transmission electron microscopy studies revealed that the density and structure of mitochondria appeared unaffected by *Bmal1* deficiency (data not shown). These observations suggest that *Bmal1* deficiency results in adult onset lipid accumulation in brown adipocytes.

**Figure 5:**
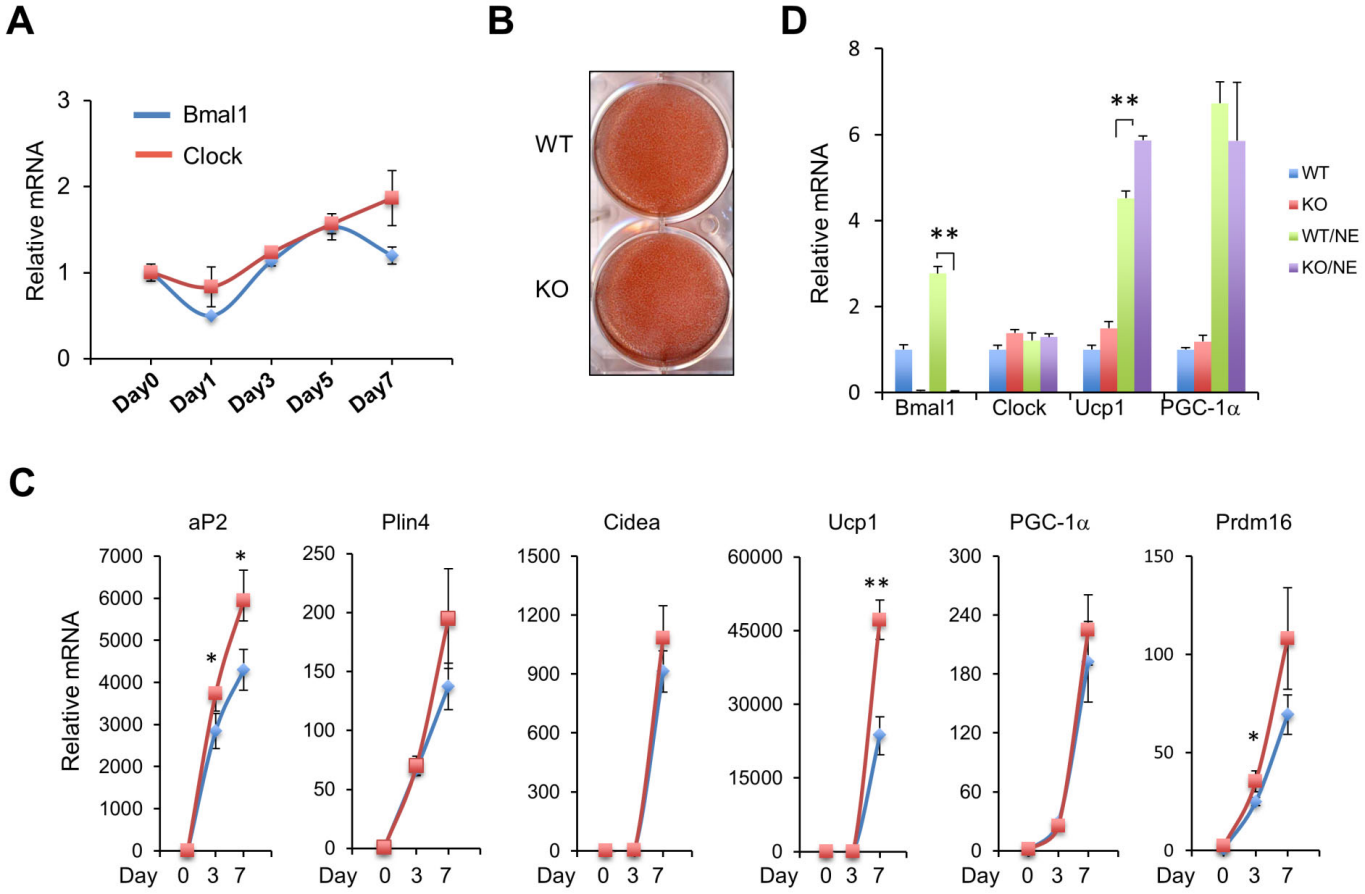
Role of *Bmal1* in brown adipocyte differentiation. A. Time course expression of Bmal1 and Clock during brown adipocyte differentiation. B. Oil Red-O staining of adipocytes following 7 days of differentiation. C. qPCR analyses of brown adipocyte gene expression during differentiation. D. qPCR analyses of brown adipocyte gene expression following treatments with vehicle or 1 µM NE for 5 hrs. Data are collected from 3 replicates and represent mean ± stdev. WT vs. KO, *p<0.05, **p<0.01.

We next examined whether *Bmal1* deficiency affects brown adipocyte differentiation in culture. Using a previously established protocol [42], we immortalized brown preadipocytes dissociated from neonatal brown fats using large T-antigen. Immortalized preadipocytes were then induced to differentiate into brown adipocytes. The latter expresses key molecular markers of brown fat and responds to β-adrenergic stimulation. The expression of *Bmal1* and *Clock* was moderately increased during the course of differentiation (Fig. 5A). Oil red-O staining on fully differentiated brown adipocytes revealed that lipid accumulation appeared similar in control and *Bmal1* deficient brown adipocytes (Fig. 5B). Gene expression analyses on brown fat markers indicated that the expression of *Plin4*, *Cidea*, and *PGC-1α* was similar between control and *Bmal1* deficient adipocytes. Expression of *Prdm16* is significantly elevated on day 3. The mRNA levels of *Ucp1*, and *aP2*, key adipogenic markers, were similarly augmented in *Bmal1* null cells three or seven days after differentiation (Fig. 5C). The expression of *Ucp1* was also higher in *Bmal1* null adipocytes following norepinephrine treatments (Fig. 5D). Together, these results suggest that, while Bmal1 is not required for the differentiation of brown adipocytes and the formation of brown adipose tissue, it is required for maintaining normal expression of genes involved in thermogenesis and lipid metabolism.

### Brown fat clock is dispensable for defense against cold exposure

**Figure 6:**
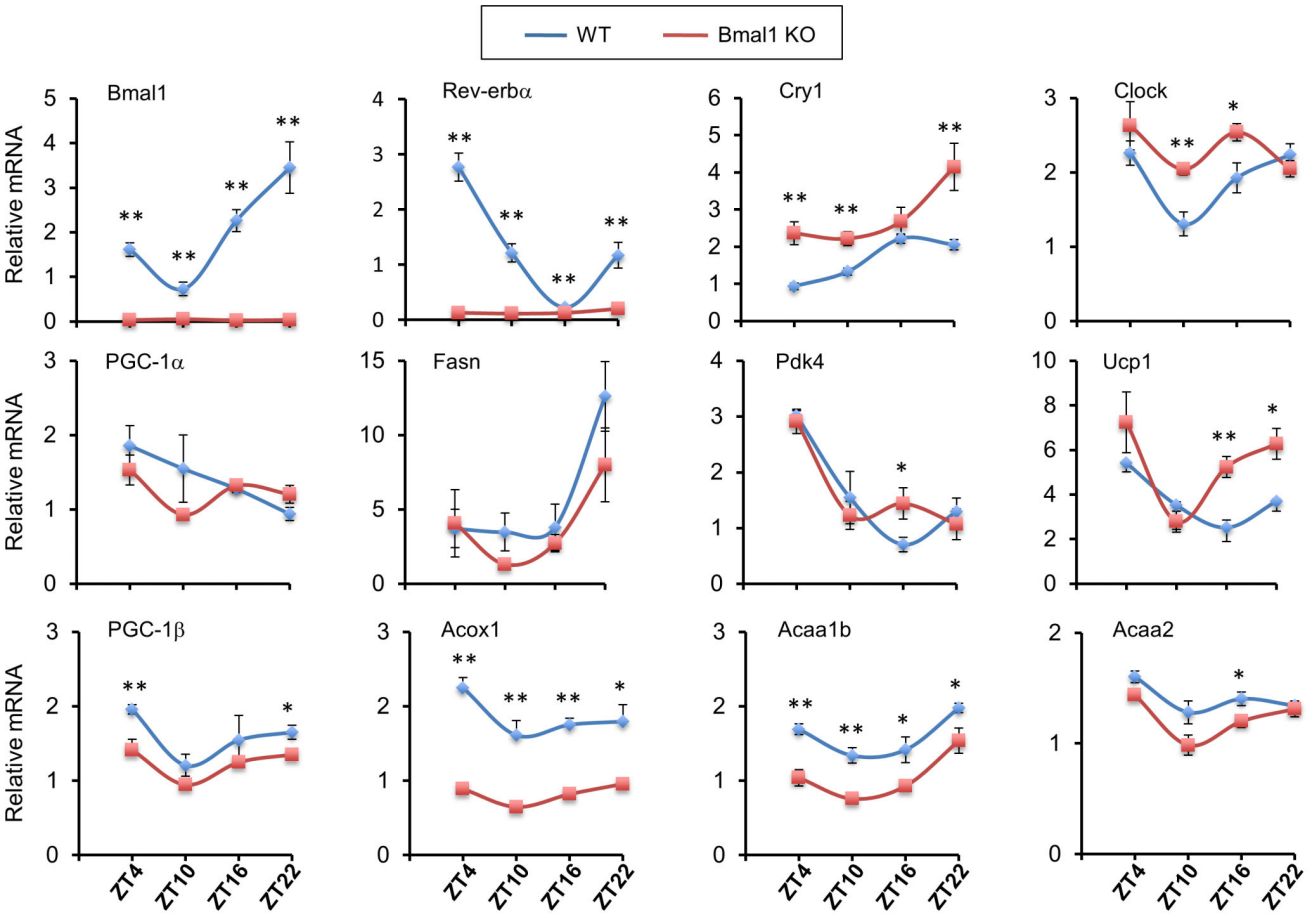
Circadian regulation of BAT gene expression. BAT from WT and *Bmal1* KO mice were collected at ZT4, 10, 16, and 22 (Zeitgeber time 0 is defined as the onset of subjective light phase; n = 4–6 mice for each data point per group). Total RNA was isolated for qPCR analyses of clock and metabolic gene expression. Data represent mean ± SEM. WT vs. KO, *p<0.05, **p<0.01.

Many metabolic enzymes display rhythmic expression patterns in the peripheral tissues according to the light/dark cycles [43], [44], [45]. To examine whether clock is required for diurnal regulation of BAT gene expression, we collected tissues and analyzed gene expression at four different time points. Similar to previous observations [46], [47], deletion of *Bmal1* resulted in marked dampening of rhythmicity of clock gene expression in BAT, including *Rev-erbα, Cry1* and *Clock* (Fig. 6). While the expression of *PGC-1α* and fatty acid synthase (*Fasn*) did not differ significantly in brown fat in two groups, we observed aberrant diurnal expression of *Ucp1* and several genes involved in fatty acid β-oxidation, such as *PGC-1β*, peroxisomal acyl-CoA oxidase 1 (*Acox1*), acetyl-CoA acyltransferase 1b (*Acaa1b*), *Acaa2*, and pyruvate dehydrogenase kinase 4 (*Pdk4*). Interestingly, *Ucp1* mRNA expression was significantly elevated at ZT16 and 22 in *Bmal1*-deficient brown fat (Fig. 6).

**Figure 7:**
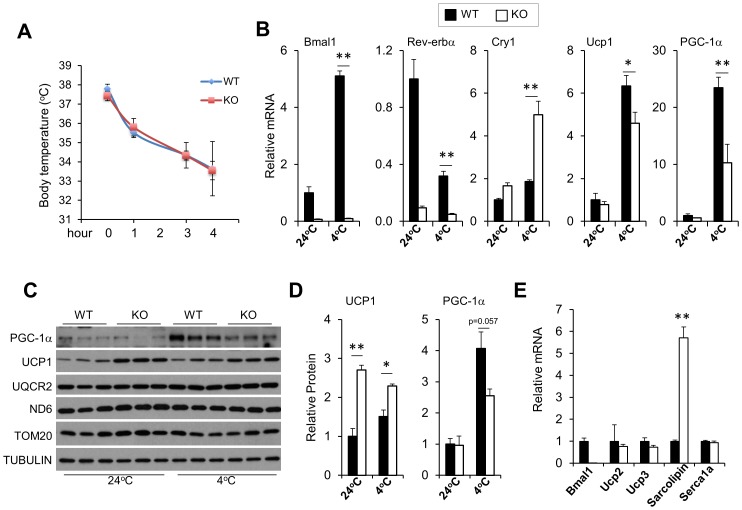
*Bmal1*-deficient mice are cold-tolerant. A. Rectal temperature in Bmal1 WT (n = 5) and KO (n = 4) mice during cold exposure. B. qPCR analyses BAT gene expression in WT (filled) and KO (open) mice at room temperature (24°C) or 5 hours after cold exposure (4°C). C. Immunoblots of total BAT lysates from treated mice. D. The UCP1 and PGC-1α protein levels were graphed from immunoblot C after normalization to TUBULIN. E. Skeletal muscle gene expression in WT (n = 4) and KO (n = 4) mice after cold exposure. Data represent mean ± SEM, WT vs. KO, *p<0.05, **p<0.01.

To determine whether brown fat clock is required for adaptive thermogenesis, we subjected control and Bmal1 null mice to acute cold exposure and measured their core body temperature. Surprisingly, *Bmal1* null mice remained cold-tolerant and were nearly indistinguishable from the control group in maintaining the core body temperature (Fig. 7A). Gene expression analyses revealed that mRNA expression of clock components *Rev-erbα* and *Cry1*, and *PGC-1α* was significantly altered in BAT from *Bmal1* null mice before and after cold exposure (Fig. 7B). Similar to mRNA, protein expression of *PGC-1α* was lower in BAT of knockout mice following cold exposure (Fig. 7C, D). The protein levels of mitochondrial proteins, ubiquinol-cytochrome c reductase core protein II (UQCR2), NADH dehydrogenase 6 (ND6), and translocase of outer membrane 20 (TOM20), on the other hand, remain similar between wild type and knockout mice (Fig. 7C). While induction of *Ucp1* mRNA expression by adrenergic signaling was significantly blunted in mice lacking a functional clock (Fig. 7B), UCP1 protein levels were elevated before and after cold (Fig. 7C, D). Interestingly, the expression of *Sarcolipin*, a factor recently found to contribute to muscle thermogenesis [48], was markedly elevated in *Bmal1* null skeletal muscle following cold exposure (Fig. 7E). In contrast, mRNA levels of *Ucp2, Ucp3*, and Sarco/endoplasmic reticulum Ca^2+^-ATPase 1 (*Serca1a*), a calcium pump involved in muscle thermogenesis, were similar between the two groups. These observations suggest that the biological clock is required for proper response to sympathetic stimulation. Despite this, the molecular clock is dispensable for defense against cold stress, likely through activating compensatory thermogenic mechanisms.

## Discussion

In this study, we demonstrated that the brown fat clock is highly responsive to cold exposure and adrenergic receptor activation. The expression of *Bmal1*, a core clock gene, was induced by cold exposure and adrenergic activation in vivo and in cultured brown adipocytes, suggesting that *Bmal1* expression may be a direct target of adrenergic signaling. We found that the expression of *PGC-1α*, a transcriptional coactivator that regulates mitochondrial biogenesis and clock gene expression, was regulated in a manner similar to *Bmal1*. It is possible that *PGC-1α*, which stimulates the transcription of *Bmal1* through coactivating nuclear hormone receptors *RORα* and *RORγ* [30], may mediate the effects of adrenergic signaling on *Bmal1* expression. Indeed, induction of *Bmal1* expression during cold stress was blunted in BAT from *PGC-1α* deficient mice. Consistently, white adipocytes failed to stimulate *Bmal1* expression in response to cold exposure, likely as a result of low *PGC-1α* and *PGC-1β* expression. Recent works have provided evidences that low amplitude temperature changes are capable of entraining the circadian oscillator [23], [24], [25], [49]. As such, the drop of core body temperature during the initial stage of cold stress might trigger the resetting of clocks through direct temperature sensing.

Previous studies have demonstrated that *Bmal1* may play a role in the differentiation of 3T3-L1 and mouse embryonic fibroblasts into white adipocytes [50], [51]. However, conflicting results were reported in these studies, likely due to the use of different adipocyte differentiation models. The relatively normal appearance of white adipose tissue in *Bmal1* deficient mice indicated that *Bmal1* is largely dispensable for lineage determination. More recently, mice with *Bmal1* deficiency specifically in adipocytes were generated and found to develop obesity due to increased food intake [47]. As such, it appears that *Bmal1* may serve critical roles in adipocyte function but is not required for the development of the adipocyte lineage. In this study, we found that brown adipocytes from mice lacking *Bmal1* had enlarged lipid droplets. *Bmal1* appeared to be dispensable for differentiation of immortalized brown preadipocytes in culture. While the density and morphology of mitochondria in brown adipocytes appeared similar between two groups, cold-inducible expression involved in adaptive thermogenesis was impaired in brown fat from *Bmal1* null mice. The expression of clock genes and genes involved in fatty acid β-oxidation was also significantly perturbed, indicating that *Bmal1* is required for the activation of the thermogenic gene program in brown fat. BAT and WAT have recently been shown to originate from distinct precursor pools during development. It is possible that *Bmal1* may serve different roles in the development and function of white and brown adipocytes, similar to previous observations of cell-specific effects of clock in the liver and pancreas [52], [53].

To our surprise, *Bmal1* deficient mice exhibited no notable defects in defense against cold exposure and were able to maintain core body temperature. Compared to control, *Bmal1* null mice were smaller in size and consequently had larger surface/body weight ratio [54]. As such, it is likely that thermogenic demand for maintaining body temperature may be elevated in Bmal1 null mice. Interestingly, *Ucp1* mRNA and protein levels were elevated in *Bmal1* deficient brown fat at ZT16 and ZT22. In vitro studies in cultured adipocytes indicate that this increase in *Ucp1* expression is likely mediated through a cell-autonomous mechanism. In addition, mRNA expression of *Sarcolipin* in skeletal muscle from *Bmal1* null mice was elevated, suggesting that compensatory mechanisms, particularly muscle-based non-shivering thermogenesis, were induced to accommodate the demand for increased thermogenic capacity.

An important unanswered question is the physiological significance of the crosstalk between adrenergic signaling and the biological clock. It is possible that adrenergic tone impinges on the molecular clock in BAT, which in turn modulates adaptive thermogenesis and the maintenance of energy balance. Indeed, as discussed above, adipocyte-specific deletion of *Bmal1* exacerbates diet-induced obesity in mice [47]. The significance of brown fat clock in mediating these effects, however, remains to be elucidated. Alternatively, brown fat thermogenesis may play a role in diurnal regulation of core body temperature. Recent studies suggest that the fluctuation of body temperature itself may serve as a zeitgeber in synchronizing different body clocks [23], [24]. As such, the brown fat clock may serve to synchronize clock oscillators in other tissues through its regulation of heat production and body temperature.

## Materials and Methods

### Ethics Statement

All animal procedures were approved by the University Committee on Use and Care of Animals at the University of Michigan. The approval number is PRO00001482.

### Mice

*Bmal1* knockout mice were obtained from the Jackson Laboratory (stock #009100) and maintained by breeding between heterozygous mice. Mice were fed *ad lib* and maintained under 12/12 h light/dark cycles. Zeitgeber time 0 is defined as the onset of subjective light phase. For histology, brown fat was fixed in 4% Formaldehyde overnight, embedded in paraffin, sectioned, and stained with haematoxylin and eosin. For cold exposure, at ZT5, 6-week old mice were individually housed in cages pre-chilled at 4°C. Mice have free access to food and water during the experiments. Core body temperature was monitored using a rectal thermometer at indicated time points. At ZT10, 5 hours after cold challenge, brown fat was dissected and immediately frozen in liquid nitrogen before RNA and protein analyses. For propranolol treatments, saline or propranolol (3 mg/kg) were administered to 9-week old male mice by intraperitoneal injection. Twenty minutes later, mice were subjected to cold exposure for 3 hours before tissue collection. For CL-316,243 treatments, 12-week old male mice were injected daily with saline or CL-316,243 (i.p., 1 mg/kg) for 7 days.

### Brown adipocyte differentiation

Primary brown adipocytes were immortalized as previously described [42]. Briefly, brown adipose tissues from wild type and *Bmal1* knockout neonates were removed and dissociated using type I collagenase. The cells were transduced with a retrovirus expressing large T-antigen and selected in the presence of G418 (400 µg/ml). Stably transduced cells were subsequently used in differentiation studies. For differentiation, confluent cells were cultured in DMEM supplemented with 10% FBS containing 0.5 mM IBMX, 125 µM Indomethacin, 2 µM dexamethasone, 1 nM T3 and 20 nM insulin. 48 hours later, the induction medium was replaced with maintenance medium (DMEM supplemented with 10% FBS, 1 nM T3 and 20 nM insulin). Fresh maintenance medium was added every 2 days until day 7. For the time course differentiation studies, the cells were harvest on different day for RNA analysis as indicated. Oil Red-O staining was performed 7 days after differentiation. Briefly, cells were fixed in 2% formaldehyde for 10 minutes. After washing with ddH_2_O twice, the cells were stained for 1 hour with 0.2% Oil red-O diluted from 0.3% stock solution in ddH_2_O. The cells were then stored or scanned after 2 brief wash with ddH_2_O.

### Gene expression analyses

Total RNA from tissues and cultured cells was extracted using TRIzol method following manufacturer instructions. For quantitative real-time PCR (qPCR) analysis, equal amount of RNA was reverse-transcribed using MMLV-RT followed by quantitative PCR reactions using SYBR Green (Life Technologies). Relative mRNA abundance of each gene was normalized to ribosomal protein 36B4 and/or beta-actin. The sequences of qPCR primers were listed in Table S1. Student's t-test was performed to assess statistical significance.

For immunoblotting studies, total protein lysates were prepared from BAT by homogenization in lysis buffer (50 mM Tris-HCl, pH 7.8, 137 mM NaCl, 10 mM NaF, 1 mM EDTA, 1% Triton X-100, 10% glycerol, and protease inhibitors). The lysate was then cleared by centrifugation. Equal amounts of total protein were analyzed on SDS-PAGE followed by immunoblotting using rabbit anti-UCP1 (Alpha Diagnostic), rabbit anti-Tom20 (Santa-Cruz), mouse anti-OXPHOS antibody cocktail (MitoSciences), and rabbit anti-PGC-1α.

## Supporting Information

Table S1 **List of qPCR primers used in this study.**
(XLSX)
